# Use and Misuse of Emergency Room for Children: Features of Walk-In Consultations and Parental Motivations in a Hospital in Southern Italy

**DOI:** 10.3389/fped.2021.674111

**Published:** 2021-06-08

**Authors:** Maria Calicchio, Francesco Valitutti, Antonio Della Vecchia, Anna Giulia Elena De Anseris, Lucia Nazzaro, Sara Bertrando, Dario Bruzzese, Pietro Vajro

**Affiliations:** ^1^Clinical Pediatrics and Pediatrics, University Hospital “San Giovanni di Dio e Ruggi d'Aragona”, Salerno, Italy; ^2^Medical Administration, University Hospital “San Giovanni di Dio e Ruggi d'Aragona”, Salerno, Italy; ^3^Department of Preventive Medical Sciences, Federico II University, Naples, Italy; ^4^Pediatrics Section, Department of Medicine, Surgery and Dentistry “Scuola Medica Salernitana”, University of Salerno, Baronissi, Italy

**Keywords:** pediatric emergency department, non-urgent visits, parental perception of urgency, primary care, ED overcrowding

## Abstract

**Objective:** Inappropriate use of the emergency department (ED) represents a major worldwide issue both in pediatric and adult age. Herein, we aim to describe features of pediatric visits to the ED of Salerno University Hospital and to evaluate parental reasons behind the decision to walk in.

**Materials and Methods:** We performed a retrospective observational study evaluating ED encounters for children from January 2014 to December 2019. The appropriateness of visits was measured with a national tool assessing every ED encounter, namely, “the Mattoni method,” which consists of the combination of the triage code assigned, the diagnostic resources adopted, and the consultation outcomes. Moreover, 64 questionnaires were collected from a sample of parents in the waiting rooms in January 2020.

**Results:** A total number of 42,507 visits were recorded during the study period (19,126 females; mean age ± *SD*: 4.3 ± 3.8 years), the majority of whom were inappropriate (75.8% over the considered period; 73.6% in 2014; 74.6% in 2015; 76.3% in 2016; 76.7% in 2017; 77.9% in 2018; 75.5% in 2019). Most of the inappropriate consultations arrived at the ED by their own vehicle (94.4%), following an independent decision of the parents (97.2%), especially in the evening and at night on Saturdays/Sundays/holidays (69.7%). A multivariate analysis revealed the following: patients of younger age (OR: 1.11, 95% C.I. 1.06–1.16; *p* < 0.0019), night visits (OR 1.39; 95% C.I.: 1.32–1.47; *p* < 0.001), patients living in the municipality of Salerno (OR 1.28; 95% C.I.: 1.22–1.34; *p* < 0.001), weekend day visits (OR 1.48; 95% C.I.: 1.41–1.56; *p* < 0.001), and independent parental decision without previous contact with primary care pediatrician (OR 3.01; 95% C.I.: 2.64–3.44; *p* < 0.001) were all significant independent predictors of inappropriate consultation. The most frequent trigger of ED encounters was fever (51.4%). Hospital admission made up 17.6% of all consultations. The questionnaire showed that most parents were aware of the lack of urgency (20.3%) or minor urgency (53.1%) of the visit. The reasons for walking in were the impossibility to receive a home consultation (70%), the difficulty of contacting their family pediatrician during weekends and holidays (54.4%), as well as the search for a quick, effective, diagnosis and therapy (48.4%).

**Conclusions:** The study suggests a highly inappropriate use of ED for children in our region. This issue deserves considerable attention by health care system leaders in order to optimally integrate hospitals and primary care.

## Introduction

An ED (Emergency Department) is the part of a hospital dedicated to recently injured or sick people who need prompt treatment. Notwithstanding its distinctive role, through the years this area has witnessed a high increase of unnecessary visits that should instead be dealt with by other appropriate primary care services, ideally available and easily reachable in our communities. This unsteady balance between unnecessary primary care consultations wrongly addressed in the ED and available resources reveals an overcrowding condition into emergency rooms, which negatively affects both the patients (through a collateral increase in waiting time, lack of confidentiality, and efficiency) and hospital staff (the higher risk of burnout, lower motivation, and professional satisfaction, increase in violence episodes toward health care professionals for long waits) (1, 2). This condition is widespread in several countries around the world and it affects both the pediatric and general ED. International studies have been conducted to analyze the inappropriate use of the ED (3, 4). In December 2003, Italian central and regional governments agreed on “Mattoni SSN” project with the aim of evaluating the appropriateness of every emergency room visit by its triage code assigned, the diagnostic resources adopted, and consultation outcomes (5).

The aims of this research were to describe the features of pediatric encounters to the ED of Salerno University Hospital and to evaluate parental reasons behind the decision to walk in. To date, no such study has been conducted in Southern Italy, and a perspective on the organizational challenges for pediatric health care of our geographic area is lacking.

## Methods and Materials

A retrospective study was performed on pediatric consecutive consultations to the ED of the San Giovanni di Dio and Ruggi d'Aragona University Hospital in Salerno during years 2014–2019. This is an Academic Hospital with an ED attended by roughly 90,000 patients yearly and providing emergency care for an urban/suburban area of 1,107,000 inhabitants. Inclusion criteria: all electronic clinical files from children aged 0–14 years seen by pediatricians. Fourteen years is the upper age limit for pediatric consultations within the ED. Exclusion criteria: pediatric surgical encounters were not part of this study because at our institution such cases are seen directly by pediatric surgeons.

The primary study outcomes for this retrospective cohort were disease severity and appropriateness, which were then analyzed by considering other variables, such as the timing of the ED visit, means of transportation, preliminary primary care referral, and outcome (discharge, hospitalization). Disease severity was considered by the initial triage code assigned according to the Emergency Severity Index version 4 (ESI v.4) (6). Appropriateness was rated according to the Mattoni method, a nationwide system of classification for appropriate encounters to ED expressed as a ratio of non-urgent patients discharged from the ED for whom no diagnostic test was required over the total of ED consultations (5). All data were extracted with our ED software. Coupled with this, a prospective and dedicated survey was carried out on a sample of parents during the first 10 days of January 2020. This period was fully homogeneous with the previous winter month and previous winter seasons with respect to the number and severity of encounters for children. This specific survey was conducted by means of a structured questionnaire proposed to parents in the waiting room. This survey was approved by the Medical Administration as part of an internal audit, alongside the retrospective branch of our study. The proposed questionnaire (Supplementary Material 1) involved several sections such as (1) the anonymized demographics of child and parents, (2) data on children's primary care pediatricians/general practitioners, (3) the reasons for going to the ED, (4) waiting times and parental satisfaction, and (5) an assessment of appropriateness by the pediatrician who performed the visit. All collected data remained anonymous throughout the study.

The questionnaire was structured according to several items considered in previous studies (7–13).

### Statistical Analysis

Statistical analysis was performed using IBM SPSS Statistics (IBM Corp., Released 2017; IBM SPSS Statistics for Windows, Version 25.0. Armonk, NY, USA) and the R platform (R Core team, 2020). Data are presented as absolute frequencies, percentage, means, and standard deviations. Univariate associations with primary outcomes (appropriateness and disease severity) were assessed with crude odds ratios (OR) with the corresponding 95% Confidence Intervals (95% CI); a multivariate logistic regression model was built to assess the independent association with those factors that showed a significant effect at univariate analysis. A value of *p* <0.05 was considered significant in all analyses.

## Results

**Figure 1:**
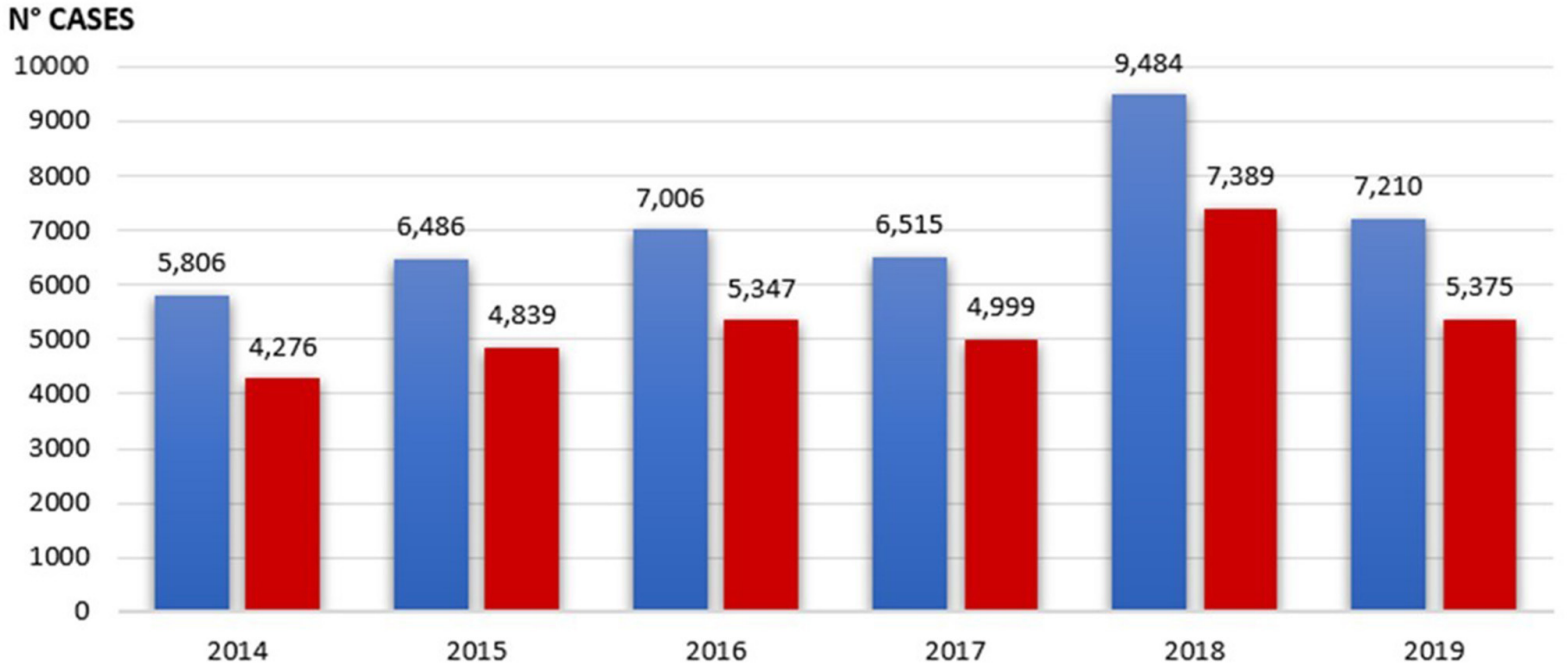
Blue columns represent total visits per year; red columns represent inappropriate visits per year.

We registered 42,507 encounters during years 2014–2019 (19,126 females; mean age ± *SD*: 4.3 ± 3.8 years), with quite steady values of inappropriate ones ranging from 73.6% (2014) to 77.9% (2018) (Figure 1). Triage codes and consultation outcomes are summarized in Table 1. The use of resources related to pediatric consultations in the ED are reported in Table 2.

**Table 1:** Outcomes of pediatric Emergency Department visits during years 2014–2019 according to triage codes.

**Outcome of ED visit**	***N***	**(%)**	***N* White triage codes**	***N* Green triage codes**	***N* Yellow triage codes**	***N* Red triage codes**
			**(%)**	**(%)**	**(%)**	**(%)**
Home discharges	23,220	54.6	1,044 (4.5%)	22,059 (95.0%)	117 (0.5%)	0 (0%)
Referral for a specialty outpatient clinic	10,089	23.7	494 (4.9%)	9,554 (94.7%)	40 (0.4%)	1 (0.01%)
Hospitalizations	7,488	17.6	265 (3.5%)	6,806 (90.9%)	381 (5.1)	36 (0.5%)
Refused hospitalizations	1,004	2.4	30 (3.0%)	939 (93.5%)	35 (3.5%)	0 (0%)
Leaving the Emergency Department before the visit	301	0.7	15 (4.8%)	277 (92.2%)	8 (2.7%)	1(0.3%)
Leaving the Emergency Department during the visit	167	0.4	14 (8.4%)	151 (90.4%)	2 (1.2%)	0 (0%)
Transfer to another center	238	0.6	7 (2.9%)	208 (87.4%)	20 (8.4%)	3 (1.3%)
Total	42,507	100	1,869 (4.4%)	39,994 (94.1%)	603 (1.4%)	41 (0.1%)

**Table 2:** Diagnostic-therapeutic resources used for children accessing to the Emergency Department (2014–2019) and mean readout/report time from physical examination (when applicable).

**Diagnostic-therapeutic resources**	**Total (%)**	**Mean readout/report time (±*SD*)**
Blood laboratory tests	4,498 (10.6%)	74 min (61–87 min)
Other specialist consultations	2,576 (6.1%)	n/a
CT scan	1,618 (3.8%)	22 min (7–37 min)
ECG	598 (1.4%)	n/a
Intravenous therapy	256 (0.6%)	n/a
Abdominal ultrasound	162 (0.4%)	39 min (28–50 min)
Echocardiography	74 (0.2%)	44 min (19–69 min)
Electroencephalography	106 (0.2%)	40 min (22–70 min)

*CT, computerized tomography; ECG, Electrocardiogram*.

Most of the emergency encounters followed independent parental decisions (97.2%), without a preliminary primary care assessment or phone consultations. Children were brought to the ED by their parents' vehicles (94.4%), especially in the evening and at night (69.7%) of holidays or dates preceding holidays (Supplementary Materials 2, 3). Table 3 summarizes reasons for ED consultation of our pediatric population classified according to ICD9-CM (14).

**Table 3:** List of discharge diagnoses^*^.

**Diseases**	**Total *N***	**(%)**
Fever	21,831	51.4
Respiratory issues	9,594	22.5
Neurologic issues	2,297	5.4
Dermatologic issues	2,891	6.7
Psychiatric issues	1,266	2.9
Gastrointestinal issues	1,148	2.8
Infectious and parasitic diseases	1,084	2.6
Endocrine, nutrition, metabolic, and immune disorders	669	1.6
Injuries and Poisoning	663	1.6
Osteomuscular issues	257	0.6
Hematological issues	202	0.5
Perinatal conditions	169	0.4
Tumors	162	0.4
Genitourinary issues	136	0.3
Circulatory system issues	101	0.2
Congenital malformations	37	0.1

* *The clinical conditions are listed according to the classes of the ICD9-CM (International Classification of Diseases ∧ 9th revision, Clinical Modification) (12)*.

The outcome of the consultations was a discharge in the majority of cases (54.6%), with a medical prescription or referral for a specialty outpatient clinic (23.7%); hospitalizations, however, accounted for only 17.6%.

No association between inappropriate visits and variables such as gender or nationality was found. Multivariate analysis revealed that patients of a younger age (OR: 1.11, 95% C.I. 1.06–1.16; *p* < 0.0019), night visits (OR 1.39; 95% C.I.: 1.32–1.47; *p* < 0.001), patients living in the municipality of Salerno (OR 1.28; 95% C.I.: 1.22–1.34; *p* < 0.001), weekend day visits (OR 1.48; 95% C.I.: 1.41–1.56; *p* < 0.001), and independent parental decisions without previous contact with a primary care pediatrician (OR 3.01; 95% C.I.: 2.64–3.44; *p* < 0.001) were all significant independent predictors of inappropriate consultation (Table 4). Arriving at the ED with one's own vehicle, significantly associated with the outcome at univariate analysis, was no longer significant in the multivariate model.

**Table 4:** Univariate and multivariate analyses of predictors of non-urgent/inappropriate visits^*^.

			**Univariate**		**Multivariate**	
	**Appropriate (*n* = 10,451; 24.6%)**	**Non-urgent/Inappropriate (*n* = 32,056; 75.4%)**	**O.R. [95% C.I.]**	***p*-value**	**O.R. [95% C.I.]**	***p*-value**
**Age**						
>5 years	3,799 (36.4)	10,583 (33)	Ref.			
≤ 5 years	6,652 (63.6)	21,473 (67)	1.16 [1.11–1.21]	<0.001	1.11 [1.06–1.16]	<0.001
**Gender**						
Female	4,703 (45)	14,423 (45)	Ref.			
Male	5,748 (55)	17,630 (55)	1 [0.96–1.05]	0.996		
**Nationality**						
Italian	10,289 (98.4)	31.536 (98.4)	Ref.			
Not Italian	162 (1.6)	520 (1.6)	1.05 [0.88–1.25]	0.621		
**Residence**						
Out of Salerno district	6,672 (63.8)	18,688 (58.3)	Ref.			
Salerno district	3,779 (36.2)	13,368 (41.7)	1.26 [1.21–1.32]	<0.001	1.28 [1.22–1.34]	<0.001
**Consultation time**						
Morning/Afternoon	7,975 (76.3)	22,618 (70.6)	Ref.			
Evening/Night	2,476 (23.7)	9,438 (29.4)	1.34 [1.28–1.41]	<0.001	1.39 [1.32–1.47]	<0.001
Weekday
Weekdays	7,519 (71.9)	20,282 (63.3)	Ref.			
Saturday/Sunday	2,932 (28.1)	11,774 (36.7)	1.49 [1.42–1.56]	<0.001	1.48 [1.41–1.56]	<0.001
**Arrived to ED by**						
Other	1,055 (10.1)	1,758 (5.5)	Ref.			
Parents' vehicles	9,396 (89.9)	30,298 (94.5)	1.94 [1.79–2.1]	<0.001	1.09 [0.98–1.22]	0.113
**Sent to ED by**						
Other	848 (8.1)	854 (2.7)	Ref.			
Parental independent decision	9,603 (91.9)	31,202 (97.3)	3.23 [2.93–3.56]	<0.001	3.01 [2.64–3.44]	<0.001

*ED, Emergency Department; O.R., odds ratio; ref., reference value*.

* *Non-urgent/inappropriate visits defined according to the Mattoni method (5). Round parentheses indicate %*.

The *post-hoc* sample of questionnaires collected (*n* = 64) expanded data on parental characterization that were not available from the ED software, such as age, education, and employment (Table 5). The questionnaire was completed by 30 mothers, 10 fathers, and both parents in 23 cases; only one questionnaire was completed by a grandmother. For all cases, the questionnaires contained demographic and social data of both parents. Most parents finished high school (mothers: 50.0%; fathers: 48.4%) and/or held a university degree (mothers: 25.0%; fathers: 17.2%), but a proportion of parents had just completed secondary schools (mothers: 21.8%; fathers: 31.3%); in addition, a very small percentage achieved only a primary school degree (mothers: 3.1%; fathers: 3.1%). Among mothers, the 50% were housewives or unemployed. On the other hand, 93.8% of fathers were employed. Eighty-nine percent of the surveyed children were regularly checked by their own primary care pediatrician, but only 86% of the parents reported that they knew his/her outpatient clinic timetables. Most of the parents (70%) declared that it was very challenging to get an appointment or even just talk over the telephone with their pediatrician on weekends or bank holidays.

**Table 5:** Demographics of caregivers regarding the access to the Emergency Department (information from questionnaires).

	**Mothers**	**Fathers**
**Average age (years)**	36	39
**Nationality**	Italian: 96.8 %	Italian: 96.8 %
	Foreign: 3.1%	Foreign: 3.1%
**Education**		
Primary school degree	2 (3.1%)	2 (3.1%)
Secondary school degree	14 (21.8%)	20 (31.3%)
High school degree	32 (50.0%)	31 (48.4%)
University degree	16 (25.0%)	11 (17.2%)

As for the urgency perceived by the parents on a scale of 1–10, parents assigned an average urgency value of 6.1 vs. a value of 3.5 judged by the pediatrician assessing their children in the ED. Most of them were aware of the non-urgency (20.3%) or minor urgency (53.1%) of the condition and declared openly that they showed up in the emergency room just for a quicker and more effective diagnosis and therapy (Supplementary Material 4).

## Discussion

This study relies on quite a large set of data collected over 6 years of observation at Salerno University Hospital. The ample temporal space of this collection and the high number of cases allowed us to portray a wide, homogeneous, accurate, and descriptive scenario of pediatric visits to ED. Inappropriate consultations, defined according to the Mattoni method (5), represent the majority of our cases (32,226 out of 42,507 cases, 75.8%). However, this indisputably high misuse of pediatric visits to our ED is not surprising and echoes even worse reports of the international literature, which reports up to 90% of inappropriate encounters elsewhere (3, 4, 7–11, 14). With respect to previous published data from Italy, our results of inappropriate encounters lay in between two previous surveys from northern Italy that described inappropriateness in 60% (12) and in 90% (13) of the cases.

Our retrospective study refers to a pre-Covid 2019 timeframe, but inappropriate and low-resource-intensity pediatric visits to ED have also been registered during this latter pandemic albeit with lower numbers (15, 16). In the light of our demographics, younger age (below 5 years) represented a constant feature of the majority of consultations, with neonates and toddlers below 12 months accounting for 20% overall, likely due to the feeling of frailty associated with this age group. Analysis of the time frame for ED pediatric encounters showed a higher number of cases during evenings and nights, but also in the days prior to bank holidays and during bank holidays; in our setting, this is most likely attributable to the lack of availability of primary-care pediatricians who are not on call on these days. It is worth noting that the pediatric health care system in Italy is part of the National Health System and comprises three main levels of intervention: first access/primary care, secondary care/hospital care, and tertiary care based on specialty hospital care (17). So far, in Italy every child aged 0–6 must be registered with a primary care pediatrician from the national health system, while for those aged 6–14, parents are allowed to choose between registration with a primary care pediatrician or with a general practitioner. Roughly 8,000 primary care pediatricians currently work nationwide, with each of them representing the primary health care provider for up to 1,000 children. Every primary care pediatrician is available at his/her office either in the morning or the afternoon for at least 3 hours per day and is reachable over the phone for at least 2 extra hours per day; primary care pediatricians are also on call on Saturday morning from 8 a.m. to 10 a.m. However, until the medical houses begin operating, pediatric primary care during night hours and weekends is not available, thus forcing families with children to seek help from the adult primary care continuity service (which usually sends them to ED) or directly from the ED. Our study endorses the need for a reorganization of the current system and also highlights the necessity of improving the parents' lack of education of children's health.

In fact, albeit limited to a small group of parents, our prospective survey results also allowed us to briefly glimpse into parental motivations and decision-making for taking their children to the ED.

It was interesting to notice that both education and employment are relevant for this. The education level is pivotal because it relates to health literacy, i.e., understanding of health needs and opportunities (WHO Health Promotion Glossary, 1998) (18, 19).

Our study highlighted that in our province a non-negligible percentage of parents coming to our ED had not achieved a high school degree and some even had attended primary school only. In order to reduce unnecessary visits to the pediatric emergency room, a look at parental motivations might be useful. As seen also in our series, the perception of the need for immediate care can possibly be attributed to a higher degree of anxiety and a distorted idea of emergency, as well as social factors or distrust of previous medical advice (12, 20–25). Other topics pertaining to the predilection for ED include the belief or hope of receiving the best quality of care because of the professionals and the diagnostic equipment at hospitals; the effortlessness of the consultation; the continuity; the rapidity in getting a diagnosis without the need of an appointment; and lack of fees (8, 12, 20, 22, 26). This preference for ED encounters by families is also sustained by the pitfalls of primary care services, such as the above-mentioned limited opening hours of general pediatricians and unavailability to carry out diagnostic tests (8, 12, 13, 20, 22, 23, 25, 27). According to our work, the reasons mentioned above are largely confirmed. In addition, despite the fact that some parents are aware of the non-urgency or minor urgency of their children's visit to the ED, the discrepancy in perceived urgency between parents and the doctor on call has still been notable. A suitable solution to contrast this phenomenon could be campaigns to improve parents' literacy regarding when it is wise and necessary to go promptly to the ED (24–26, 28–30).

In the last section of the questionnaire, parents themselves are asked for some advice to reduce unnecessary overcrowding in ED. However, only 17% of them felt they wanted more information about this issue, while the majority (50%) simply proposed increasing the number of staff in the ED since “it is the best place to treat their child and get a prompt/effective diagnosis and therapy.” This preference for the ED has also been attested to by a very recent report from Turkey that attributed this aspect to both the lack of cost and the trust in the ED staff, especially among younger and less educated parents (31). Otherwise, the second most suggested solution has been “to increase the availability of our family pediatricians and to improve availability of primary care.” In this regard, a UK study has demonstrated that increasing primary care accessibility might lessen the burden of ED visits from children (32); a similar effect can also be obtained by relocating unwarranted consultations to dedicated outpatient settings, thus relieving the overload of ED, as witnessed in a study from France (33).

It will be interesting to evaluate whether future programs of pediatric telemedicine may be of value in reducing overcrowding and inappropriate visits; for the time being, pediatric telemedicine in ED has been reported to be a suitable tool to minimize transfers from ED lacking pediatric specialists to PED (34).

Our study has some limitations that may have impacted or influenced the interpretation of the findings from this research, still leaving some questions unanswered. First, we are aware that our database does not include final diagnoses for several clinical issues that have been first evaluated in the ED and further addressed for inpatients after admission or by other specialty outpatient clinics. Moreover, we could not report the use of point-of-care testing in our retrospective cohort due to the impossibility of data extrapolation. A prospective research design would have eventually allowed us to fill these information gaps. Secondly, by a prospective questionnaire we attempted to portray the motivations that pushed some parents to take their children to the ED, although this group might not have been fully representative of the target population due to minor temporal lag, and this mixed method approach certainly restricts the generalizability of our findings. In addition, such a single center study design may suggest that our study cohort lacks generalizability on a national level; although this is methodologically plausible, to the best of our knowledge the Italian scenario for primary and hospital care for children is somewhat homogeneous.

## Conclusions

The majority of ED visits at our center were inappropriate, with a stable trend over the years. Parental reasons for inappropriate presentations most commonly reflected difficulties in contacting primary care pediatricians and the perceived need for a quick work-up and treatment. Learning also from the experience of the recent epidemic, the real challenge perhaps remains to move health care systems toward more integrated models between hospitals and community services, simultaneously, and helpfully operating on the same wavelength for our children.

## Data Availability Statement

The raw data supporting the conclusions of this article will be made available by the authors, without undue reservation.

## Ethics Statement

Ethical review and approval was not required for the study on human participants in accordance with the local legislation and institutional requirements. Written informed consent to participate in this study was provided by the participants' legal guardian/next of kin.

## Author Contributions

MC collected the data, co-analyzed the data, and co-drafted the manuscript. FV analyzed the data, co-drafted, and revised the manuscript. ADV authorized access to electronic files and edited the manuscript. AGEDA, LN, and SB organized the prospective survey, collected the data, and edited the manuscript. DB provided statistical suggestions, analyzed the data, and drafted the method section. PV conceived and designed the study, co-drafted the manuscript, is the guarantor as the senior co-author. All authors contributed to the article and approved the submitted version.

## Conflict of Interest

The authors declare that the research was conducted in the absence of any commercial or financial relationships that could be construed as a potential conflict of interest.

## Abbreviations

CT scan: computerized tomography scan
ECG: Electrocardiogram
ER: emergency room
ED: emergency department
ESI: emergency severity index.

## References

[1] Bernestein SL Aronsky D Dusejia R Epstein S Handel D Hwang U. The effect of emergency department crowding on clinically oriented outcomes. Acad Emerg Med. (2009) 6:1–10. 10.1111/j.1553-2712.2008.00295.x 19007346

[2] Abudan A Merchant RC. Multi-dimensional measurements of crowding for pediatric emergency departments: a systematic review. Glob Pediatr Health. (2021) 8:1–11. 10.1177/2333794X21999153 33718529 PMC7923972

[3] Durand AC Gentile S Devictor B Palazzolo S Vignally P Gerbeaux P. ED patients: how nonurgent are they? Systematic review of the emergency medicine literature. Am J Emerg Med. (2011) 29:333–45. 10.1016/j.ajem.2010.01.003 20825838

[4] Mistry RD Cho CS Bilker WB Brousseau DC Alessandrini EA. Categorizing urgency of infant emergency department visits: agreement between criteria. Acad Emerg Med. (2006) 13:1304–11. 10.1197/j.aem.2006.07.028 17099192

[5] Ministero della Salute - Progetto Mattoni: Obiettivi Generali. Available online at: www.mattoni.salute.gov.it/mattoni/paginaMenuMattoni

[6] Green NA Durani Y Brecher D DePiero A Loiselle J Attia M. Emergency Severity Index version 4: a valid and reliable tool in pediatric emergency department triage. Pediatr Emerg Care. (2012) 28:753–57. 10.1097/PEC.0b013e3182621813 22858740

[7] Benahmed N Laokri S Zhang WH Verhaeghe N Trybou J Cohen L. Determinants of nonurgent use of the emergency department for pediatric patients in 12 hospitals in Belgium. Eur J Pediatr. (2012) 171:1829–37. 10.1007/s00431-012-1853-y 23064744

[8] Costed Wong A Claudet I Sorum P Mullet E. Why do parents bring their children to the emergency department? A systematic inventory of motives. Int J Family Med. (2015) 2015:1–10. 10.1155/2015/978412 26618002 PMC4649091

[9] Kalidindi S Mahajan P Thomas R Sethuraman U. Parental perception of urgency of illness. Pediatr Emerg Care. (2010) 26:549–53. 10.1097/PEC.0b013e3181ea71b3 20657341

[10] MacFaul R Stewart M Werneke U Taylor-Meek J Smith H.E Smith I.J. Parental and professional perception of need for emergency admission to hospital: prospective questionnaire-based study. Arch Dis Child. (1998) 79:213–18. 10.1136/adc.79.3.213 9875015 PMC1717678

[11] Zickafoose JS DeCamp LR Prosser LA. Association between enhanced access services in pediatric primary care and utilization of emergency departments: a national parent survey. J Pediatr. (2013) 163:1389–95. 10.1016/j.jpeds.2013.04.050 23759421 PMC3796049

[12] Pecile P Pittini C Pusiol A Tenore A. Indagine Regionale Sull'accesso al Pronto Soccorso Pediatrico. Medico e Bambino Pagine Elettroniche 2000. 3. https://www.medicoebambino.com/?~id=RI0010_10.html

[13] Vedovetto A Soriani N Merlo E Gregori D. The burden of inappropriate emergency department pediatric visits: why Italy needs an urgent reform. Health Serv Res. (2014) 49:1290–05. 10.1111/1475-6773.12161 24495258 PMC4239850

[14] Center for Disease Control and Prevention (CDC). Available online at:https://www.cdc.gov/nchs/icd/icd9cm.htm#:~:text=ICD%2D9%2DCM%20is%20the,10%20for%20mortality%20coding%20started

[15] Valitutti F Zenzeri L Mauro A Pacifico R Borrelli M Muzzica S. Effect of population lockdown on pediatric emergency room demands in the era of COVID-19. Front. Pediatr. (2020) 8:521. 10.3389/fped.2020.00521 33072657 PMC7530634

[16] DeLaroche AM Rodean J Aronson PL Fleegler EW Florin TA Goyal M. Pediatric emergency department visits at US children's hospitals during the COVID-19 epidemics. Pediatrics. (2020) 147:39628. 10.1542/peds.2020-039628 33361360

[17] Corsello G Ferrara P Chiamenti G Nigri L Campanozzi A Pettoello-Mantovani M. The child health care system in Italy. J Pediatr. (2016) 177S:S116–26. 10.1016/j.jpeds.2016.04.048 27666260

[18] World Health Organization (1998) Health Promotion Glossary. Geneva: WHO. Available online at: https://www.who.int/healthpromotion/about/HPG/en/

[19] Smith BJ Tang KC Nutbeam D. WHO health promotion glossary: new terms. Health Promot Int. (2006) 21:340–45. 10.1093/heapro/dal033 16963461

[20] Berry A Brousseau D Brotanek JM Tomany-Korman S Flores G. Why do parents bring children to the emergency department for nonurgent conditions? A qualitative study. Ambul Pediatr. (2008) 8:360–67. 10.1016/jamb2008.07.001 19084785

[21] Burokiene S Raistenskis J Burokaite E Cerkauskiene R Usonis V. Factors-determining parents' decisions to bring their children to the pediatric emergency department for a minor illness. Med Sci Monit. (2017) 23:4141–48. 10.12659/MSM.902639 28845042 PMC5584823

[22] Butun A Linden M Lynn F McGaughey J. Exploring parents' reasons for attending the emergency department for children with minor illnesses: a mixed methods systematic review. Emerg Med J. (2018) 36:39–46. 10.1136/emermed-2017-207118 30389792

[23] Kua PH Wu L Ong EL Lim ZY Yiew JL Thia XH. Understanding decisions leading to nonurgent visits to the paediatric emergency department: caregivers' perspectives. Singapore Med J. (2016) 57:314–19. 10.11622/smedj.2016023 26805668 PMC4971450

[24] Morrison AK Schapira MM Gorelick MH Hoffmann RG Brousseau DC. Low caregiver health literacy is associated with higher pediatric emergency department use and nonurgent visits. Acad Pediatr. (2014) 14:309–314. 10.1016/j.acap.2014.01.004 24767784 PMC4003496

[25] Williams A O'Rourke P Keogh S. Making choices: why parents present to the emergency department for non-urgent care. Arch Dis Child. (2009) 94:817–20. 10.1136/adc.2008.149823 19395399

[26] Salami O Salvador J Vega R. Reasons for nonurgent pediatric emergency department visits: perceptions of health care providers and caregivers. Pediatr Emerg Care. (2012) 28:43–46. 10.1097/PEC.0b013e31823f2412 22193700

[27] Pileggi C Raffaele G Angelillo IF. Paediatric utilization of an emergency department in Italy. Eur J Publ Health. (2006) 16:565–69. 10.1093/eurpub/ckl019 16478806

[28] Watson M Blair M. Emergency departments and minor illness: some behavioural insights. Arch Dis Child. (2018) 103:309–10. 10.1136/archdischild-2017-314057 29317439 PMC5890632

[29] DeAngelis C Fosarelli P Duggan AK. Use of the emergency department by children enrolled in a primary care clinic. Pediatr Emerg Care. (1985) 1:61–5. 10.1097/00006565-198501020-00004 3843434

[30] Morrison AK Myrvik MP Brousseau DC Hoffmann RG Stanley RM. The relationship between parent health literacy and pediatric emergency department utilization: a systematic review. Acad Pediatr. (2013) 13:421–29. 10.1016/j.acap.2013.03.001 23680294 PMC3808118

[31] Kurt F Begde F Oguz S Tekin D Suskan E. How important are parental age and educational level in nonurgent admissions to the pediatric emergency department? Pediatr Emerg Care. (2020) 36:414–18. 10.1097/PEC.0000000000001886 31464877

[32] Cecil E Bottle A Cowling TE Majeed A. Pediatrics. (2016) 137:e20151492. 10.1542/peds.2015-1492 Wolfe I, Saxena S. Primary care access, emergency department visits, and unplanned short hospitalizations in the UK 26791971

[33] Demonchy D Haas H Gillet Vittori L Montaudie I Piccini-Bailly C Tran A. A short track to relieve pediatric emergency department overload. Arch Pediatr. (2015) 22:247–54. 10.1016/j.arcped.2014.12.013 25612876

[34] Varma S Schinasi DA Ponczek J Baca J Simon NE Foster CC. A retrospective study of children transferred from General Emergency Departments to a Pediatric Emergency Department: which transfers are potentially amenable to telemedia-ne? J Pediatrics. (2021) 230:126–32. 10.1016/j.jpeds.2020.10.070 33152370

