# Supplementary material for: Use and Misuse of Emergency Room for Children: Features of Walk-In Consultations and Parental Motivations in a Hospital in Southern Italy
Source: Front Pediatr. 2021 Jun 8;9:674111. doi: 10.3389/fped.2021.674111 (PMC8217610; doi:10.3389/fped.2021.674111)
Supplement: Supplementary file 1 [file Table_1.DOCX]

**Supplementary 1:**  Parents' questionnaire regarding the access to the ED

**DATE: ___/____/________ TIME:__________**

**TRIAGE CODE ASSIGNED:** □White □ Green □Yellow □Red

**SECTION 1: PERSONAL DATA**

**Child:**

1. Gender of the patient: **□** Male; **□** Female;
2. Age: ______
3. Nationality: ________
4. Residence: □ Salerno; □ Salerno Province; □ Other:_________

**PARENT/CAREGIVER:**

1. Type of kinship with the child: □ Mother; □ Father; □ Both; □ Other:______
2. Age mother: ______; age father: ________;
3. Nationality of parents: __________
4. Highest school degree obtained:

- Mother: □ Primary school degree ; □ Secondary school degree; □ High school degree; □ University degree;
- Father: □ Primary school degree; □ Secondary school degree; □ High school degree; □ University degree;

1. Employment:

- Mother: ________________
- Father:________________

1. Number of children: _______

**SECTION 2: INFORMATION ON THE PRIMARY CARE DOCTOR**

1. Your son/daughter is followed by:

□ General Pediatrician

□ General Practioner (without pediatric specialty)

□ Other: _________

1. Your child is regularly followed-up by:

□ Periodic well-being checks

□ Follow-ups for known chronic disease: _____________

□ Urgent consultations when needed

□ Prescription of drugs and examinations

1. How many times in the last 12 months has he/she been to the doctor: ______
2. Are you aware of the opening hours for the office of your child's doctor during weekdays?

□ Yes □ No Time: __________________

1. What is the availability during holidays?_______________________________________
2. Is it challenging to have an appointment with your doctor? □Yes □No
3. Waiting time for an appointment: ______ days
4. In case of urgency is it possible to be received without an appointment? □Yes □No
5. Does the doctor go to your house if necessary? □Yes □No
6. Does your doctor answer to phone requests?

□ Only during working hours

□ Yes, at any time during weekdays

□ Always also during holidays

□ No.

1. Does the doctor have a secretary/assistant filtering phone calls? □Yes □No

**SECTION 3: REASONS FOR ACCESS TO THE EMERGENCY ROOM**

1. The decision to go to the ED:

□ was autonomous

□ recommended by the Pediatrician

□ recommended by another doctor

□ recommended by the emergency service

Other ____________

1. Reasons behind access to ED:

□ Search for a diagnose and rapid/effective treatment

□ I consider the ED the best place to treat my son

| □ Proximity (Minutes from home by car:____) |
| --- |

□ We are responsible parents

□ Seen by the general pediatrician on the same day, not satisfied

□ I could not contact the general pediatrician

□ General pediatrician is not available for home consultation

□ The working hours of my general pediatrician are not comfortable

□ Brought to ED by Ambulance

Other ____________

1. Please indicate:

The severity of the urgency perceived by the family for the condition of the child:

□ There's no urgency. The child needs checks or therapy already established;

□ Urgency is less. The child complains of symptoms, which do not affect heart, respiratory or neurological functions, but needs treatment;

□ there is urgency. The patient has a partial impairment of the functions of the circulatory or respiratory system, any alterations in consciousness, complains of intense pain; there is no immediate danger of life, but it needs to be monitored by the doctor as soon as possible;

□ There is extreme urgency. Danger of life.

- What you consider serious, from 1 to 10, the health condition of your child:


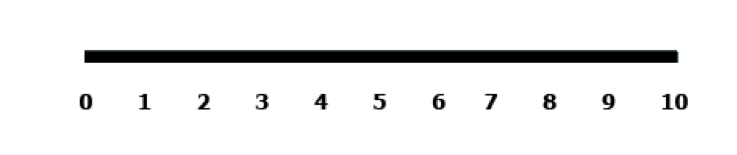


1. Reason for the visit:

□ Fever

□ Dehydration

□ Rashes

□ Respiratory issues

□ Gastrointestinal issues (nausea, vomiting, diarrhea, abdominal pain, etc...)

□ Incessant Crying

□ Osteomuscolar issues

□ Neurological issues

□ Known Chronic Pathology

□ Intoxication

□ Other: _______________

1. How long has the child manifested the problem? □ Hours □ Days

**SECTION 4: EMERGENCY ROOM**

1. Waiting time for the visit: _________
2. Are you satisfied with the services provided by our ED? □Yes □No
3. If your answer is NO, why? ______________________________
4. Is this the first time you have visited the ED for the child's health? □Yes □No
5. If your answer is NO, indicate:

- how many times has there been in the previous year: _______
- which ED he went to: _________
- the assigned code: □ white □ green □ yellow □ red

1. Would you return to ED if your child were to re-present the same health? □Yes □No
2. What do you recommend to reduce pediatric PS crowding?

□ to increase staff (doctor/nurse)

□ to give parents more guidance on how and when to turn to ED

□ to create a phone network available during public holidays and at night for advice and guidance

□ to expand the availability of work for family pediatricians

□ to organize a external pediatric continuity of care that covers public holidays and night hours

Other_____________________________________________________________

**SECTION 5: FOR THE PEDIATRICIAN WHO VISITED THE CHILD**

Please indicate:

1. The severity of the child just visited through a numerical scale from 1 to 10 (1 minimum value-10 maximum value):


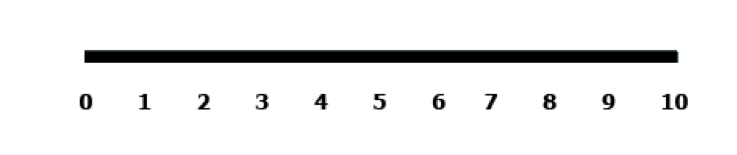


2. The appropriateness or otherwise of the visit in a context of ED:

□ Inappropriate

□ Appropriate

3. Exit Triage Code:

□ White

□ Green

□ Yellow

□ Red

4. Outcome of visit:_____________
