# Supplementary material for: Use and Misuse of Emergency Room for Children: Features of Walk-In Consultations and Parental Motivations in a Hospital in Southern Italy
Source: Front Pediatr. 2021 Jun 8;9:674111. doi: 10.3389/fped.2021.674111 (PMC8217610; doi:10.3389/fped.2021.674111)
Supplement: Supplementary file 2 [file Table_2.DOCX]

**Supplementary 2:** Access modality to Emergency Department (2014 – 2019)

| **ACCESS MODE** | **N°** | **(%)** |
| --- | --- | --- |
| **ARRIVED TO EMERGENCY DEPARTMENT BY:** |  |  |
| Ambulance | 1,474 | 3.5% |
| Parents’ vehicles | 39,695 | 93.4% |
| Not detected | 1,338 | 3.2% |
| **SENT TO EMERGENCY DEPARTMENT BY:** |  |  |
| Parental independent decisions | 40,808 | 96.0% |
| General pediatrician | 89 | 0.2% |
| Local emergency service | 1,336 | 3.1% |
| Specialist doctor | 96 | 0.2% |
| Another structure | 37 | 0.1% |
| Other | 141 | 0.3% |
| **TOTAL** | 42,507 | 100% |
