# Supplementary material for: Use and Misuse of Emergency Room for Children: Features of Walk-In Consultations and Parental Motivations in a Hospital in Southern Italy
Source: Front Pediatr. 2021 Jun 8;9:674111. doi: 10.3389/fped.2021.674111 (PMC8217610; doi:10.3389/fped.2021.674111)
Supplement: Supplementary file 3 [file Table_3.DOCX]

**Supplementary 3**. Cross-data regarding Emergency Department access time-frame during the whole study period

| **Day** | **N Accesses** | **N Patients / 24 h (mean)** | **Morning**  **N (%)** | **Afternoon**  **N (%)** | **Night**  **N (%)** |
| --- | --- | --- | --- | --- | --- |
| Monday | 5,936 | 19 | 6.2 (32.6%) | 7.3 (38.4%) | 5.5 (28.9%) |
| Tuesday | 5,242 | 16.7 | 5.3 (31.7%) | 6.4 (38.3%) | 5 (29.9%) |
| Wednesday | 5,378 | 17.2 | 5.2 (30.2%) | 7 (40.7%) | 5 (29.1%) |
| Thursday | 5,384 | 17.2 | 5.2 (30.2%) | 6.9 (40.1%) | 5.1 (29.7%) |
| Friday | 5,862 | 18.7 | 5.7 (30.5%) | 7.5 (40.1%) | 5.5 (29.4%) |
| Saturday | 7,041 | 22.5 | 7.4 (32.9%) | 9.2 (40.9%) | 5.9 (26.2%) |
| Sunday | 7,664 | 24.5 | 8.6 (35.1%) | 9.7 (39.6%) | 6.2 (25.3%) |
