# Supplementary figures and images for: Use and Misuse of Emergency Room for Children: Features of Walk-In Consultations and Parental Motivations in a Hospital in Southern Italy
Source: Front Pediatr. 2021 Jun 8;9:674111. doi: 10.3389/fped.2021.674111 (PMC8217610; doi:10.3389/fped.2021.674111)

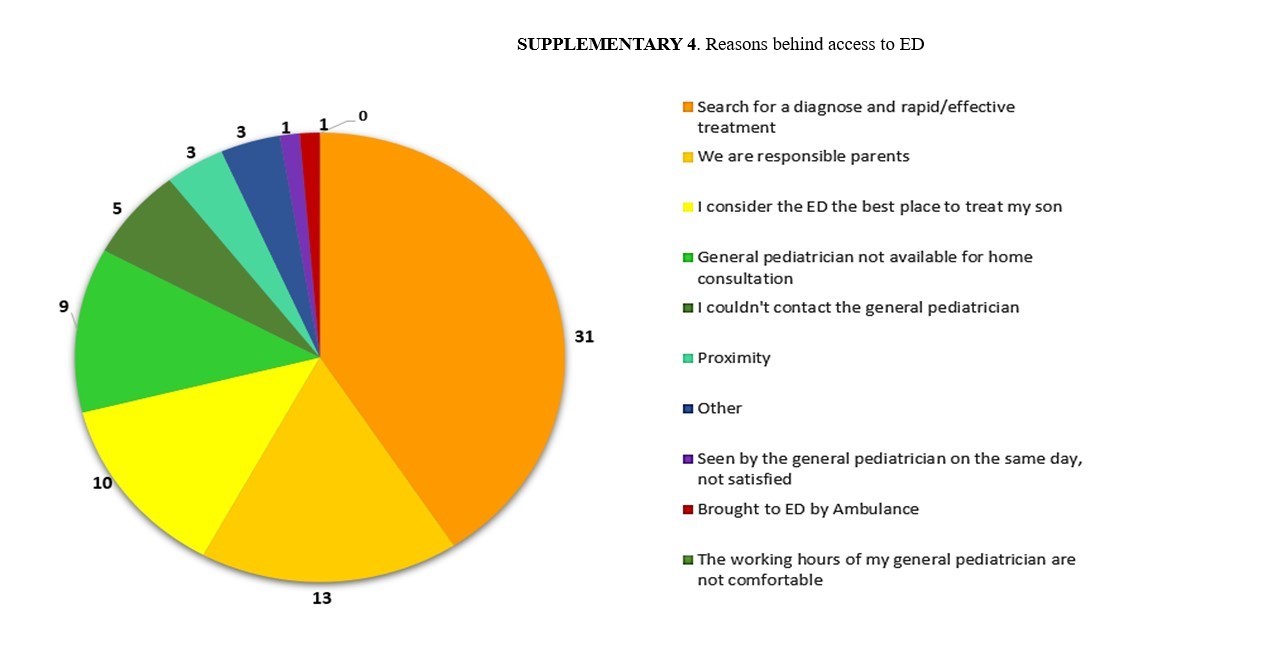

Supplement: Supplementary file 4 [file Image_4.jpg]
